# Increasing saltiness of salts (NaCl) using mid‐infrared radiation to reduce the health hazards

**DOI:** 10.1002/fsn3.3342

**Published:** 2023-04-19

**Authors:** Thangaraju Umakanthan, Madhu Mathi

**Affiliations:** ^1^ Gokulam Annadhan Temple Complex Theni Dt Tamil Nadu India; ^2^ Allianz Services Private Limited, Technopark Trivandrum Kerala India

**Keywords:** 2–6 μm of mid‐IR, economy, edible salts, health, MIRGA, potentiation, resource, saltiness, savings

## Abstract

Table salt, rock salt, and iodized salts (composed principally of NaCl) are commonly used in many areas, such as medicine, cooking, industry, or personal care. Common fried, salty, and spicy foods contain an excess of added salt, which has detrimental health effects, especially on the kidneys. Our research aims to enhance the inherent saltiness of these three salts, which would reduce intake and thereby minimize salt's health hazards. We invented a water‐based 2–6 μm of Mid‐Infrared Generating Atomizer (MIRGA), which, when applied to salts, caused changes in the salts' chemistry and enhanced saltiness, thus allowing the reduction of salt intake by 25%–30%. This easy‐to‐use technology did not show any side effects. MIRGA was found to have enhanced the saltiness, thus allowing the reduction of salt intake by 25%–30%. MIRGA is safe, portable, highly economical, unique in the mid‐IR laser technology, and possesses vast research scope in other areas of food science.

## Graphical abstract

MIRGA is a highly economical, easy‐to‐use, and safe technology in which a water‐based solution is contained in a pocket‐sized 20‐mL capacity atomizer. During spraying over the salt packets from a distance of 0.25–0.50 m, 2–6 μm mid‐infrared is generated which causes chemical bond alteration in the salt molecules. This resulted in 25%–30% increased saltiness, thereby the salt use for cooking can be cut down by 25%–30% than the usual.

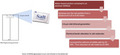

## 1 INTRODUCTION

Sodium chloride (NaCl) is a primary source of sodium and chloride ions, vital to mediate sensory reflexes. Iodized salt is enriched with iodine salts and its intake is recommended to overcome/avoid iodine deficiency (McNeil, 2006; The Lancet, 2008). Out of 84 elements in the human body, 72 are essential and also present in rock (Himalayan) salt (Shukla & van der Zee, 2010). An excess of salt intake through the consumption of modern foods causes various irreparable health issues, which is a multifaceted scientific challenge as the saltiness, quantity of salt intake, and associated diseases are directly proportional. Most people consume around twice the recommended maximum salt level, and 2.5 million deaths could be prevented yearly if global salt consumption is reduced to the recommended level (WHO, 2016). Through this research, by applying 2–6 μm of mid‐IR, we increased the saltiness of edible salts and advocated reducing the intake quantity of edible salt to prevent salt‐induced morbidity. Available literature suggests that no study has been done on potentiation and concurrent quantity reduction of edible salts.

## 2 MATERIALS AND METHODS

### 2.1 MIRGA

**FIGURE 1:**
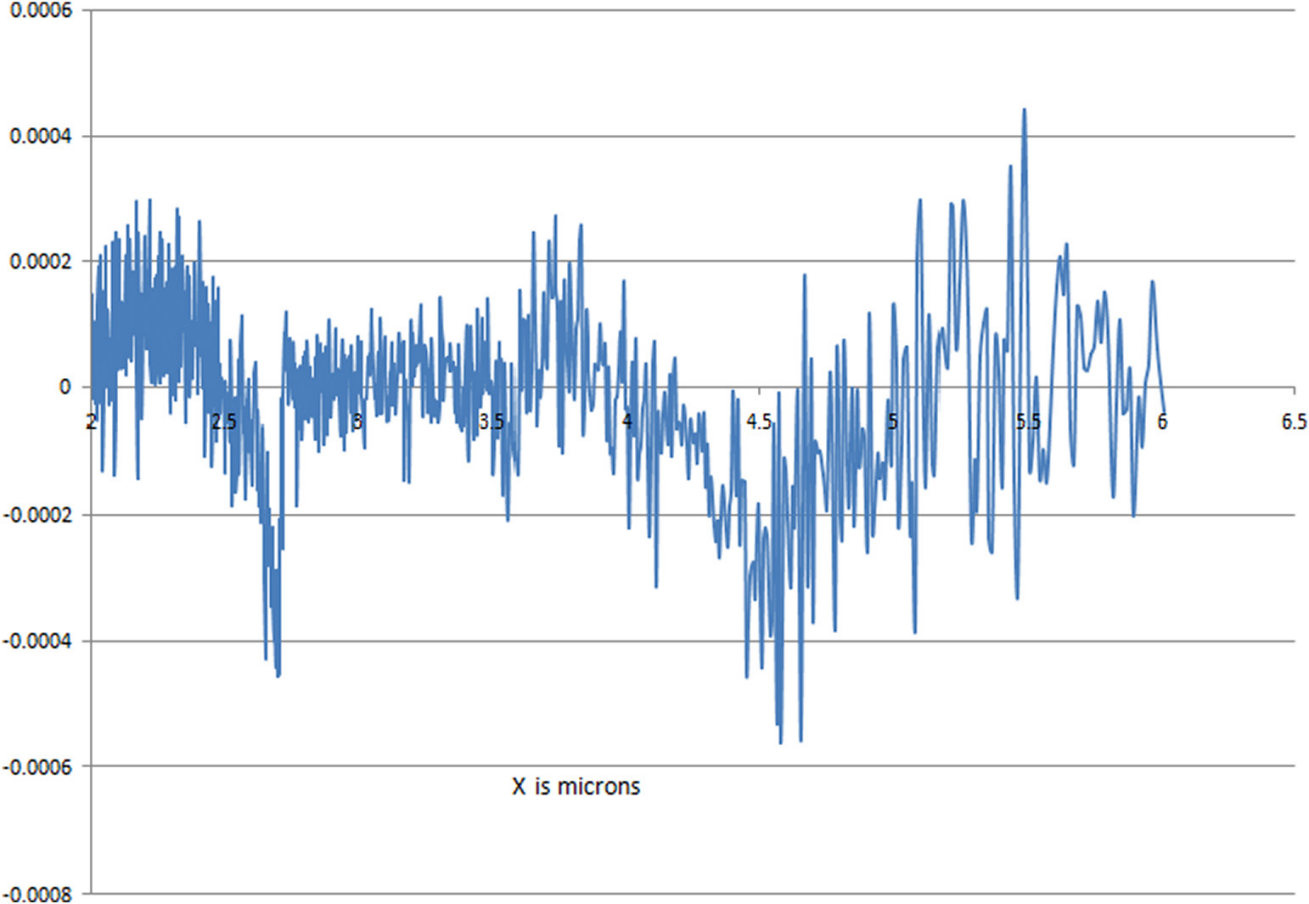
Estimation of 2–6 μm of mid‐infrared while spraying MIRGA atomizer: Spraying the MIRGA from a distance of 0.25–0.50 m toward the target substance generates infrared energy. Estimation using FTIR (retro‐reflector) interferometer instrument (Detector type D* [cm HZ1/2–1] MCT [2‐TE cooled]) at Lightwind, Petaluma, California shows that the generated energy lies in 2–6 μm of mid‐IR range.

MIRGA (*under‐patent no.: 401387*) is a 20‐mL pocket‐sized atomizer containing inorganic water‐based solution. During spraying, depending on pressure (vary with the user) applied to plunger, every spraying is designed to generate 2–6 μm of mid‐IR. Design of the MIRGA and the estimation of emitted 2–6 μm of mid‐IR (Figure 1) have been presented in detail by Umakanthan and Mathi (2022a).

### 2.2 Spraying method

Thirteen aliquots of 50 g were taken from a 1000 g batch of commercially available table salt. The aliquots were packaged in polyethylene bags with a thickness of more than 50 μm, and sealed with tape. One packet was marked “C” (control) and the remaining were numbered from 1 to 12. Corresponding to the packet number, 1 to 12 MIRGA sprayings were applied from 0.25‐ to 0.50‐m distance over the packets as one or two sprayings on one or both sides. The control packet received no spraying. Twelve sprayings had earlier been found to cause a complete loss of saltiness in the samples.

The 13 packets were individually subjected to evaluation by a sensory expert panel (Klarenbeek et al., 2014) consisting of six trained people, by consumer panel (cooks), by tasting the salt alone and also in cooked food. The results were recorded and compared. This procedure was also followed using rock salt and iodized salt. The moisture analyses were determined using the Loss on drying (LOD) method, which works according to the thermo‐gravimetric principle. The Mettler Toledo's Hydrogen Moisture analyzer (HE73 230 V Model) was used for that purpose.

Instrumental tests were performed. Chemical and structural transformation was monitored by gas chromatography–mass spectrometry (GC–MS), Fourier‐transform infrared spectroscopy (FTIR), powder X‐ray diffraction (PXRD), and transmission electron microscopy (TEM), and their details are as follows:

*GC–MS*: Agilent technologies, 7820 GC system, 5977E MSD, Column DB‐5, Over temp 100–270°C, Detector MS, Flow rate 1.2, Carrier gas Helium.

*FTIR*: IR AFFINITY I – FTIR Spectrophotometer, FTIR 7600, Shimadzu.

*PXRD*: D8 Advance (Bruker). Cu Kα radiation. High‐speed wide‐angle Lynx eye detector. Small‐angle scan for mesoporous materials can be collected from 2‐theta = 0.5°.

*TEM*: Model JEM‐2100; Make: JEOL; HT—200 kV. Sample preparation procedure: A small amount (~1 mg) of powdered sample was dispersed in the Isopropanol solvent, and the dispersion was then loaded over the TEM grid (lacey carbon‐coated copper grid—300 mesh) for analysis. Sample‐loaded dry grids were used for TEM analysis, and images were captured from different regions at 200 kV acceleration voltage.

### 2.3 Trial details

The acceptability index used by the employed sensory expert panel was the hedonic scale with a 9‐point nominal structure: 1—Dislike extremely, 2—Dislike very much, 3—Dislike moderately, 4—Dislike slightly, 5—Neither like nor dislike, 6—Like slightly, 7—Like moderately, 8—Like very much, 9—Like extremely (Everitt, 2009; Wichchukit & O'Mahony, 2014). The sprays were applied to the test sample packets as described in the previous section. The test samples were sprayed on one side and sensory tests were performed, followed by two sprayings (one on either side) and another sensory test. The number of sprayings on the packet was incrementally augmented, followed each time by a sensory test. The same trials were performed with various branded table salts, rock salts, and iodized salts. The trials were repeated six times to determine the accuracy of the tests. For each salt, three samples (control, saltiness increased, and unpleasant taste) were subjected to various instrumental tests and cooking procedures. Source of these three samples are same, but the difference between them was only the number of sprayings they received.

## 3 RESULTS AND DISCUSSION

### 3.1 Sensory expert panel test results

Comparing the sensory scores of control sample with rest of the sprayed samples, four spraying samples of table salt, rock salt, and iodized salt have been scored ‘8’ (like very much—in terms of the saltiness). Excessive number of sprayings beyond 4 has caused a gradual reduction in the sensory score. Also, the four sprayed samples has significantly reduced moisture than the control. Increased sensory qualities and reduced moisture enhanced the saltiness preferably.

Table 1 shows that the control sample is considered to have a normal taste and saltiness. In the trial samples, as the number of sprayings was increasing, the saltiness was also gradually found to be increased. After fourth spraying, the samples became more palatable than control. After the 12th spraying in table salt, 10th spraying in rock salt, and 12th spraying in iodized salt, saltiness was increased but samples became unpalatable, with an irritating and pungent taste. In the case of the cooked samples, salt requirement was 25%–30% less in the salts sprayed four times. These sensory attribute changes were perceived in 1–2 minutes after spraying, and the enhanced sensory characteristics of trialed samples were found to be retained for 18–24 months. The retention of sensory characters was conducted using accelerated stability conditions where products were stored under stress conditions. The stability of samples was then predicated using the relationship factor, the acceleration factor, and the degradation rate. The sensory panel experts’ and nontrained experts’ (cooks) opinions were almost similar except for a negligible difference.

**TABLE 1:** Moisture content and sensory scoring of salt samples.

No. of spraying	Moisture content (%)	Hedonic scale point		
		Table salt	Rock salt	Iodized salt
Control	1.38	5	5	5
1	1.09	6	6	6
2	2.01	6	6	7
3	0.71	7	7	7
4	0.68 (reduced moisture)	8 (enhanced saltiness)	8 (enhanced saltiness)	8 (enhanced saltiness)
5	1.80	7	7	7
6	0.62	6	5	6
7	0.58	5	3	5
8	0.20	4	3	4
9	0.47	4	2	3
10	1.44	3	1	2
11	0.98	2	‐	2
12	1.34	1	‐	1

### 3.2 Instrumentation results

Raw data and detailed interpretations for each salt are available, respectively, in Supplementary Data D1 and Supplementary Text T1.

#### 3.2.1 Table salt

##### GC–MS

**FIGURE 2:**
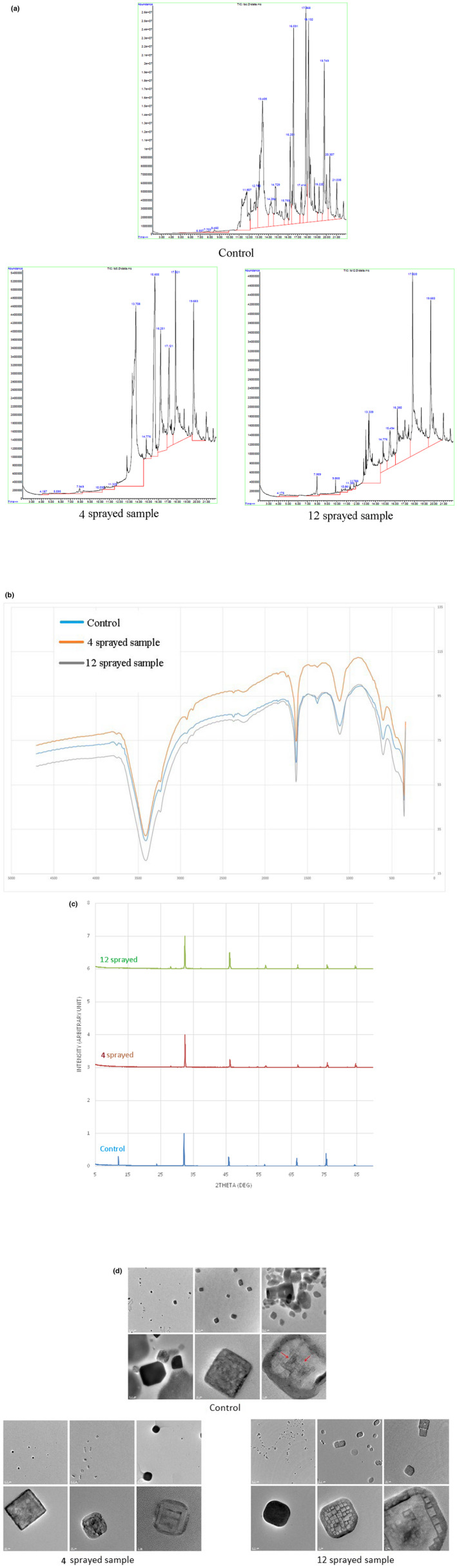
(a) GC–MS of Table salt: Numerous peaks with major ones at around 11.8, 12.8, 13.4, 14.3, 14.7, 15.7, 16.2, 16.6, 17.4, 17.9, 18.1, 19.2, 19.7, 20.3, and 21.0 min are present in control. Some of these peaks are missing in the 5 and 12 sprayed samples. Four sprayed samples have additional peaks at 13.7 and 17.1 min. Twelve sprayed samples have additional peaks at 7.9, 9.8, and 15.5 min. (b) FTIR spectra of Table Salt: Peak at 3410 cm^−1^ corresponding for OH groups water (in crystalohydrates) content is increased in 12 sprayed samples. Peak at 1635 cm^−1^ indicates that the distinct presence of scissor peak of a water molecule (–OH stretch) decreased in four sprayed samples and increased in 12 sprayed samples. Peak at 1018 cm^−1^ decreased in four sprayed samples and increased in 12 sprayed samples. (c) PXRD spectra of Table Salt: Control has NaCl_3_ phase, in contrast with 4 and 12 sprayed samples. Varying peak intensities were observed among the three samples, but no shifting of peaks was observed. Peak at around 46.0° is highest in 12 sprayed samples followed by control and the four sprayed samples. (d) TEM of Table salt: Control sample particles range from 100 to 500 nm. Four sprayed samples show 50–200 nm. In 12 sprayed samples, there are numerous particles ranging from 10 to 50 nm. In control, particles are amorphous, whereas in 4 and 12 sprayed samples, the particles are crystalline in structure.

*Control*: numerous peaks with major ones at around 11.8, 12.8, 13.4, 14.3, 14.7, 15.7, 16.2, 16.6, 17.4, 17.9, 18.1, 19.2, 19.7, 20.3, and 21.0 min. *Four sprayed samples*: compared to control, peaks are missing at around 11.8, 12.8, 14.3, 16.6, 17.4, 17.9, 18.1, and 19.2 min. There are additional major peaks at 13.7 and 17.1 min. It is suggested that the missing peaks and additional peaks give rise to the enhanced properties of the four sprayed samples. *Twelve sprayed samples*: relative to the GC–MS pattern of the control sample, the 12 sprayed samples show peaks at 7.9, 9.8, and 15.5 min and appear to be missing at 11.8, 12.8, 14.3, 15.7, and 19.2 min. These apparent differences in the GC–MS pattern are related to the strong, more pungent, undesirable taste (Figure 2a).

##### FTIR

Peak at 3410 cm^−1^ corresponds to OH groups water (in crystalohydrates) content in the sample. This peak is increased in 12 sprayed samples by 28% than control and four sprayed samples. Peak at 1635 cm^−1^ indicates the distinct presence of scissor peak of a water molecule (–OH stretch), this peak is decreased in four sprayed samples by 3%; however, in 12 sprayed samples, an increase of this peak by 36% can be noticed, which caused an unpleasant taste. Peak at 1018 cm^−1^ arises from the presence of silicates (SO_3_^2−^), which decreased by 1% in four sprayed samples and increased by 26% in 12 sprayed samples, which also caused an unpleasant taste (Figure 2b).

##### PXRD

Control shows more prominent and minor peaks among the three samples. It also has NaCl_3_ phase present, in contrast with 4 and 12 sprayed samples. Structure of 4 and 12 sprayed samples are closer compared to control, due to the presence of other underlying phases. Varying peak intensities are also observed. Peak at around 46.0° is the highest in the 12 sprayed samples, relative to the 100% peak, followed by control and in the last place, the four sprayed samples. No shifting of peaks is observed among the three samples (Figure 2c).

##### TEM

*Control*: Particle size range is 100–500 nm and an amorphous structure is observed. *Four sprayed samples*: Particle size range is 50–200 nm and crystalline structure is observed. *Twelve sprayed samples*: Particles seem to be more numerous and their size range is 10–50 nm, and crystalline structure is observed. Interplanar distance has narrower variability indicating an enhanced regular crystalline structure (Figure 2d).

The degree of crystallinity and the regularity of the crystal structure are enhanced proportionally with increasing number of MIRGA sprayings. The orientation of crystal lattice planes becomes increasingly regular and parallel to the incident electron beam, making visible the columns of atoms of the crystal lattice by increasing the number of MIRGA sprayings.

#### 3.2.2 Rock salt

##### GC–MS

**FIGURE 3:**
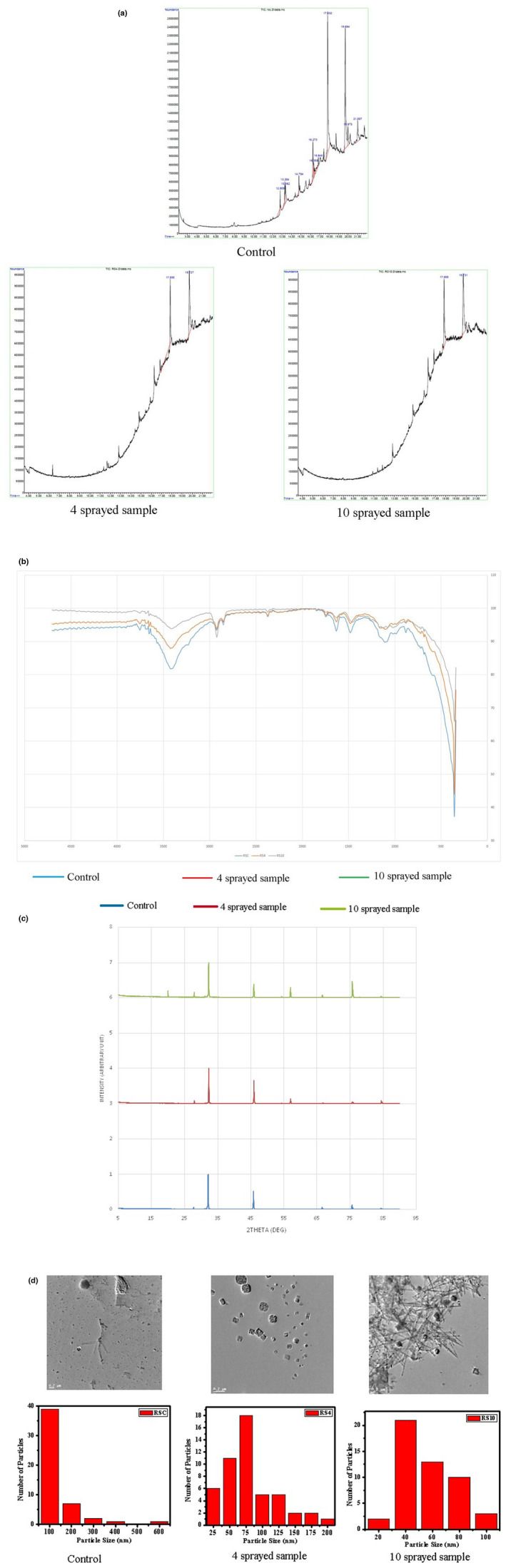
(a) GC–MS of Table salt: Peak at 17.830 Octane, 2‐cyclohexyl‐ and at 19.693 9‐Octadecenoic acid (Z)‐, 2‐hydroxy‐1‐(hydroxymethyl)ethyl ester are present only in control sample. Similarly, four sprayed samples have specific peaks at 17.855 Undecylenic acid and 19.727 6‐Octadecenoic acid, (Z)‐. Ten sprayed samples also have specific speaks at 17.85513‐Octadecenal, (Z)‐ and 19.727 Z‐8‐Pentadecen‐1‐ol acetate. (b) FTIR of Rock salt: Peak at 3410 cm^−1^ indicating the presence of water content (in crystalohydrates), peak at 1627 cm^−1^ for fluorides and peak at 1481 cm^−1^ for carbonates are decreased in 4 and 10 sprayed samples than in the control, whereas peak at 2924 cm^−1^ for bicarbonates and peak at 1018 cm^−1^ for silicates are increased in 4 and 10 sprayed samples. (c) PXRD of Table salt: Major differences between the control and sprayed samples are noticed in the peaks around 46.0°, 75.0°, 28.0°, 55.0°. Ten sprayed samples have the most number of visible peaks and extra peak at 20.1° which is absent in the control and four sprayed samples. Shifting of the most prominent peak to higher 2‐theta value is observed in both sprayed samples. (d) TEM of Rock salt: All the three samples show difference in particle size and shape. Control has nonuniform shaped particle of average size of ~138 nm. Four sprayed samples have cuboid‐shaped particles of average size of ~81 nm and 10 sprayed samples show nano‐needle‐shaped particle with an average diameter of ~55 nm.

The rock salt sample contains many aldehyde and long‐chain fatty acids. After four sprayings, the saltiness is increased and the long‐ and short‐chain fatty acids were found. More precisely, the long‐chain fatty acid (C18, 6‐Octadecenoic acid) might have broken down to medium chain (C11, undecylenic acid) during spraying. When the rock salt sample was sprayed 10 times, there were found the major peak of pentadecen‐1‐ol acetate and 13‐octadecenal. These both could be the by‐products of long‐chain fatty acid degradation and transformation (Figure 3a).

##### FTIR

Peak at 3410 cm^−1^ indicates the presence of water content (in crystalohydrates) in the control. This peak decreased for 2‐fold in the four sprayed samples and 2.7‐fold in the 10 sprayed samples. Peak at 2924 cm^−1^ indicates the presence of bicarbonates; this peak is increased by 3% in the four sprayed samples and 136% in the 10 sprayed samples, which caused unpleasant taste. Peak at 1627 cm^−1^ originates from fluorides; this peak decreased by 46% and 48% in the four and the 10 sprayed samples, respectively. Peak at 1481 cm^−1^ comes from carbonates, and decreased by 35% and 33% in the 4 and the 10 sprayed samples, respectively. Peak at 1018 cm^−1^ arises from silicates, and increased five times and seven times in the 4 and the 10 sprayed samples, respectively (Figure 3b).

##### PXRD

The 10‐time sprayed sample has the most number of visible peaks. There are six prominent peaks observed in this sample's XRD. The peak around 46.0° has highest intensity in the four‐time sprayed sample. This is followed by control and 10 sprayed samples. Peak at around 75.0° is highest at the 10 times sprayed sample, followed by control sample and the four times sprayed samples. Peak at around 28.0° is highest in the 10 times sprayed sample, followed by four times sprayed sample and the control samples. Peak at around 55.0° is highest in 10 times sprayed, followed by the four‐time sprayed sample. This peak is absent in the control sample. The 10‐time sprayed sample has one more peak at 20.1° which is absent in both control sample and 4‐time sprayed samples. Shifting of the most prominent peak to higher 2‐theta value is observed in both sprayed samples (Figure 3c).

##### TEM

Sprayings altered the shape of the particles. *Control*: Particles display a nonuniform shape with an average size of ~138 nm. *Four sprayed samples*: Cuboid‐shaped particles are present with an average particle size of ~81 nm. *Ten sprayed samples*: Particles show nano‐needle shape with an average diameter of ~55 nm (Figure 3d).

#### 3.2.3 Iodized salt

##### GC–MS

**FIGURE 4:**
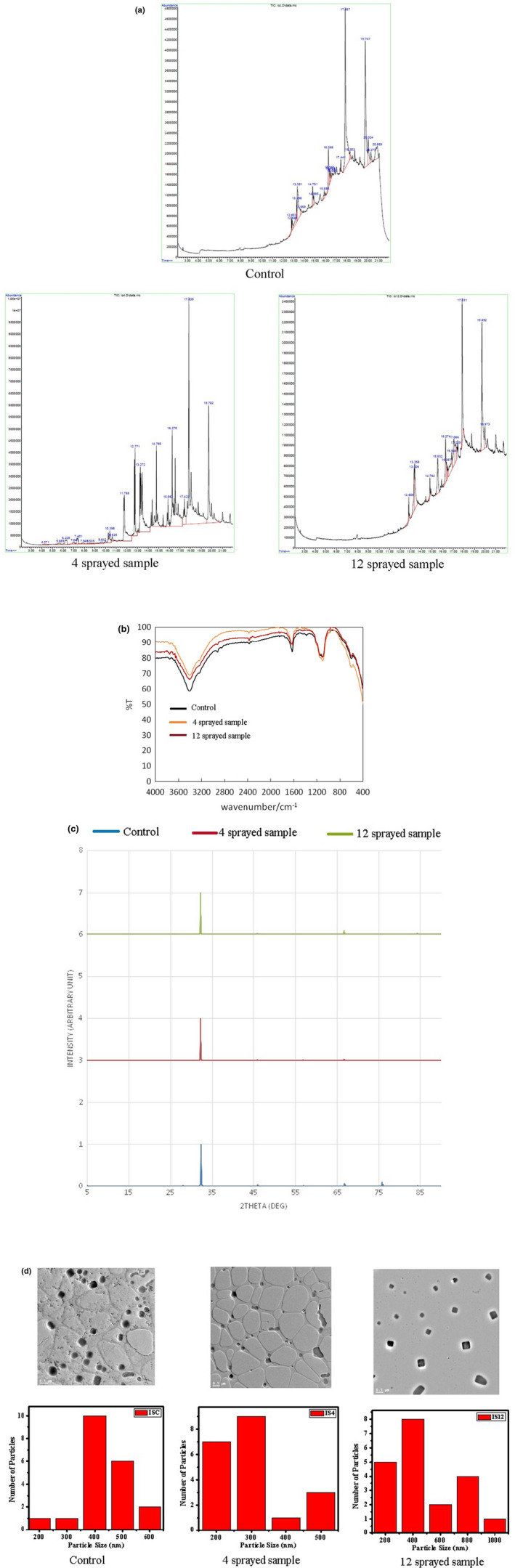
(a) GC–MS spectra of Iodized salt: Control has major peaks at around 12.8, 13.3, 14.7, 16, 17.4, 17.8, 19.7, 20, 20.2, and 20.9 min. Four sprayed samples show additional peaks at around 10.4, 10.6, and 11.8 min, and 12 sprayed samples show additional peaks at 15.5 and 16.2 min. (b) FTIR spectra of Iodized salt: All three samples have broad signals in the range of 1050–1200 cm^−1^ associated with the S=O stretching vibration of sulfate in iodized salt and another signal in the range of 3200–3600 cm^−1^ associated with the stretching vibration of O–H. Compared to control, the former peak is increased in both the 4 and 10 sprayed samples, whereas the latter peak shows no change in the four sprayed samples but is reduced in the 10 sprayed samples. (c) PXRD spectra of Iodized salt: Control has only one prominent peak located at 32.3° and visible minor peaks at 46.0°, 57.1°, 66.7°, and 75.7°. The 4 and 10 sprayed samples show only one prominent peak at 32.1°. There are variations in minor peaks of both the sprayed sample than in the control. However, the particles in all the three samples are of cubic and good crystalline structure with minimal amorphous phases. (d) TEM of Iodized salt: Control sample particles are spherical with poor crystallinity. The four sprayed sample particles are irregular, and poorly crystalline with some having leaf‐like structure. The 12 sprayed samples have well‐arranged cubic‐shaped particles with patterned structure and higher degree of crystallinity.

*Control*: it shows major peaks at around 12.8, 13.3, 14.7, 16, 17.4, 17.8, 19.7, 20, 20.2, and 20.9 min. It is suggested that the typical taste arises from compounds detected by GC–MS. *Four sprayed samples*: it shows a different chromatogram than the control sample. The key differences are additional peaks at around 10.4 min (Cyclohexane, 1,1′‐(1,3‐propanediyl)bis), 10.6 min (1,13‐Tetradecadiene), and 11.8 min (n‐Hexadecanoic acid). It is suggested that these additional peaks may be related to the increased saltiness of the four sprayed samples relative to the control sample. *Twelve sprayed samples*: There are additional key peaks at 15.5 and 16.2 min compared to the control sample. These additional peaks are due to compounds that alter the taste of the 12‐time sprayed sample, making it pungent and undesirable (Figure 4a).

##### FTIR

*Control*: The broad signal in the range of 1050–1200 cm^−1^, seen amongst others in the fingerprint region, is associated with the S=O stretching vibration of sulfate in iodized salt (Socrates, 2004). Furthermore, the broad signal in the range of 3200–3600 cm^−1^, in the functional group region, is associated with the stretching vibration of O–H^7^. *Four sprayed samples*: In the fingerprint region, in addition to a shift in the background signal, the S=O stretching vibration at 1050–1200 cm^−1^ increases by around 6%. In the functional group region, however, there is no change in the stretching vibration of O–H at 3200–3600 cm^−1^. *Twelve sprayed samples*: In the fingerprint region, the S=O stretching vibration at 1050–1200 cm^−1^ slightly drops, but it is still around 2% higher than the control signal. In the functional group region, there is now a signal drop of around 4% in the stretching vibration of O–H at 3200–3600 cm^−1^, hence depreciating the O–H bond (Figure 4b).

The average transmission signal increased in the four sprayed samples by 5.62%. In the 12 sprayed samples, the average transmission drops again, but it is still higher than the control sample by an average of 2.98%. Therefore, assuming no significant irregularities exist in the measurements, the spraying process reduces the average mid‐infrared absorption in the sample. The observed changes in the fingerprint region may be interpreted as the four‐time sprayed sample being more favorable than the control sample and the 12‐time sprayed sample (due to higher S=O stretching vibration).

##### PXRD

*Control*: it shows only one prominent peak located at 32.3°. Visible minor peaks are located at 46.0°, 57.1°, 66.7°, and 75.7°. The present peaks indicate the presence of cubic structure, close to the mineral halite (RRUFF, Halite R070292, n.d.; Almeida & Jenkins, 2017; Walker et al., 2004; Wang & Reeber, 1996). The XRD pattern shows good crystalline structure with minimal amorphous phases. Split peaks are observed in minor peaks at 46.0°, 66.7°, and 75.7° indicating departure from the standard cubic crystal structure (Figure 4c).

*Four sprayed samples*: it shows only one prominent peak located at 32.1°. Visible minor peaks are located at 46.4°, 57.5°, 66.1°, 76.2°, and, 84.6°. The present peaks indicate the presence of cubic structure, close to the mineral halite (RRUFF, Halite R070292, n.d.; Almeida & Jenkins, 2017; Walker et al., 2004; Wang & Reeber, 1996). The XRD pattern shows good crystalline structure with minimal amorphous phases. However, minor peaks are fairly discernible.

*Twelve sprayed samples*: it shows only one prominent peak located at 32.1°. Minor peaks are located at 46.4° and 66.7°. The present peaks indicate the presence of cubic structure, close to the mineral halite (RRUFF, Halite R070292, n.d.; Almeida & Jenkins, 2017; Walker et al., 2004; Wang & Reeber, 1996). The XRD pattern shows good crystalline structure with minimal amorphous phases. However, minor peaks are fairly discernible.

Control and four‐time sprayed samples have several visible minor peaks that correspond to halite with cubic crystal structure. Twelve‐time sprayed samples have only three visible minor peaks. Shifting of peaks to lower 2‐theta is observed for 4‐time and 12‐time sprayed samples.

##### TEM

Sprayings altered the shape and crystallinity of the samples. *Control*: it displays spherical‐shaped particles with an average particle size of ~435 nm and poor crystallinity. *Four sprayed samples*: It shows irregular spherical‐shaped particles with an average particle size of ~300 nm, not well arranged, and some particle shape changed to a leaf‐like structure and poor crystallinity. *Twelve sprayed samples*: it displays cuboid shaped with an average particle size of ~480 nm and well arranged, patterned structure, pure material, and higher degree of crystallinity (Figure 4d).

### 3.3 Salt quantity intake/use result

The MIRGA sprayed table, rock, and iodized salts were brand/batch‐wise used individually in cooking, dietary consumption, admixturing with other edible ingredients, in which the requirement was found to be 25%–30% lower compared to the nonsprayed control.

To summarize, all the instrumentations illustrated the effect of 2–6 mid‐IR in the salts' chemistry: chemical bond alteration, compound transformation, structure, and configuration changes that lead to the enhanced or decreased inherent characters to our desire depending on the number of MIRGA sprayings applied.

The particle size of salt crystals significantly impacts the saltiness perceived and the saltiness onset time. The researcher has shown that a smaller crystal size fraction can achieve a greater maximum saltiness per unit of sodium consumed. They have been explained by differential dissolution kinetics and enhanced mass transfer of sodium across the saliva (Rama et al., 2013). Additionally, Hurst et al. (2021) have shown that the maximum perceived saltiness can be achieved by redesigning the salt particles which include small particle size, low density, and hydrophobicity. They used a range of model salt particles to explore the impact of particle design on adhesion to product, loss in‐pack, rate of dissolution, and saltiness perception and ultimately generated a series of design rules to optimize saltiness perception.

### 3.4 Future benefits

Future benefits of the sprayed salt are as follows:

- 25%–30% reduction of required quantity, hence economy.
- Minimization of toxic health issues
- Conservation of natural resources

### 3.5 MIRGA and action of the emitted 2–6 μm of mid‐IR on salt samples

Invention background, definition, technique of mid‐IR generation from MIRGA, toxicological study on MIRGA, safety of the MIRGA sprayed usables, and primeval and future scope of MIRGA have been described by Umakanthan and Mathi 2022 (Umakanthan & Mathi, 2022a) (detailed discussion available in Supplementary Text T2).

While spraying MIRGA, most of the mid‐IR energy scatters through the air and gets absorbed by the salt molecules. Virtually all organic compounds absorb mid‐IR radiation which causes a change in molecule's vibrational state to move from the lower ground state to excited higher energy state (Girard, 2014). This leads to changes in chemical bonds (Mohan, 2004; Shankar, 2017) and these bond parameter changes lead to consequent changes in target's physical and chemical characters, configuration, and compound transformation depending on the dose of energy (Atkins & Paula, 2011; Datta et al., 2014; Yi, 2012) applied.

WHO recommends 25% salt reduction in food. On contrary, we are consuming sodium chloride more than needed. Salt directly relates to sensory attributes (Thomas‐Danguin et al., 2019). Sodium chloride concentration directly influences food matrix and sensory perception (Guichard, 2002). Usually reduced level impart negative impact on preference even though vary with food type (Aaslyng et al., 2014; Rodbotten et al., 2015). Salt inhibits bitter taste and hence, reduced salt inhibits food's bitterness (Keast & Breslin, 2002) and many methods were tried to overcome the drawbacks (Dotsch et al., 2009; Hoppu et al., 2017; Jaenke et al., 2017), among which cross‐model interaction is the better method to enhance the salty taste. The limitations of the method are addition of chemical odorants, influence of food composition on aroma, release of salt, and ultimately saltiness perception., It took 7 years to achieve 15% salt reduction in UK salt reduction program (Yeung et al., n.d.).

MIRGA technology overcame the said limitations. The spraying done on the packaged salt potentiated the saltiness, thereby salt addition to food preparation is reduced by 25%–30%. This method is affordable, easy, imminent, safe, and practicable from salt producer to user level. The method of action of 2–6 μm of mid‐IR absorbed by the water molecules of the salt causes changes in the nanostructural layer of water which leads to chemical bond alterations (as demonstrated), causing alteration of physicochemical characters, hence enhancement of saltiness (Agarwal et al., 2014; Datta et al., 2014; Esmaeili, 2015; Pollack, 2015; Sommer et al., 2008; Sommer et al., 2011; Tsai & Hamblin, 2017; Umakanthan & Mathi, 2022b). First‐time spraying is enough to retain the improved saltiness.

## 4 CONCLUDING REMARKS

MIRGA increased saltiness, leading to 25%–30% reduction in the quantity intake, thereby possibility of minimizing health hazards. Through future research by altering the MIRGA's present specifications, it would be possible to continue to reduce salt usage by further enhancing the salts' inherent characteristics (saltiness). For patients with high blood urea nitrogen, high creatinine, and renal diseases, MIRGA‐sprayed salt with various degrees of saltiness can be manufactured and suitably advocated.

## AUTHOR CONTRIBUTIONS

Conceptualization (equal); methodology (equal); project administration (equal); resources (equal); supervision (equal); validation (equal); writing – review and editing (equal). **Madhu Mathi:** Data curation (equal); investigation (equal); visualization (equal); writing – original draft (equal).

## FUNDING INFORMATION

This study received no specific funding.

## CONFLICT OF INTEREST STATEMENT

In accordance with the journal's policy and our ethical obligation as researchers, we, the authors, are reporting that we together are the inventors and patentee of Indian patent for MIRGA (*under‐patent no.: 401387*) which is a major material employed in this study.

## Supporting information

Appendix S1

Data S1

## Data Availability

All data are available in the manuscript and supplementary materials.
