# Supplementary material for: Increasing saltiness of salts (NaCl) using mid‐infrared radiation to reduce the health hazards
Source: Food Sci Nutr. 2023 Apr 19;11(6):3535–49. doi: 10.1002/fsn3.3342 (PMC10261731; doi:10.1002/fsn3.3342)
Supplement: Supplementary file 1 — Appendix S1 [file FSN3-11-3535-s001.doc]

Hedonic scale sensory evaluation questionnaire

Age:

Gender:

Date:

Overall

Aftertaste

Texture

Bitter

Sour

Sweetness

Aroma

Color

760

259

＼

Scoring standard

1- Dislike extremely, 2- Dislike Very Much, 3- Dislike Moderately, 4- Dislike Slightly, 5- Neither Like nor Hate, 6- Like Slightly, 7- Like Moderately, 8- Like

Very Much, 9- Like extremely

3Please evaluate the color and aroma from top to bottom, and evaluate the sweetness, sour, bitter, textt1re, after taste, and overall, from left to right.

4.Odor dispense is water, please drink the water (or dispense) to remove the

aftertaste and wait for 10 seconds to continue with the next sample

I.

Please taste the following sample from left to right evaluate tl1e color, aroma. sweetness, sour, bitter, texture, aftertaste, and overall, by 9-point scoring method (Integer, could repeat the score)

2. Each sample was 20 ml/ cup.

Instruction:

Suggestions:
