# Supplementary material for: Increasing saltiness of salts (NaCl) using mid‐infrared radiation to reduce the health hazards
Source: Food Sci Nutr. 2023 Apr 19;11(6):3535–49. doi: 10.1002/fsn3.3342 (PMC10261731; doi:10.1002/fsn3.3342)
Supplement: Supplementary file 2 — Data S1 [file FSN3-11-3535-s002.docx]

**Increasing saltiness of salts (NaCl) using mid-infrared radiation to reduce the health hazards**

Umakanthan^1*^, Madhu Mathi^2^

*^1*^Veterinary Surgeon, Gokulam Annadhan Temple Complex, Plot no.: 1684, Meenavilakku-Meenakshipuram Road, Anaikaraipatty Post, Bodinayakanur Taluk, Theni Dt, Tamil Nadu, India - 625582*

*^2^Veterinary claims expert, Allianz Services Private Limited, Technopark, Trivandrum, Kerala – 695581, India.*

**Corresponding author: Ph.09345572218; Email:* [*rkbuma@gmail.com*](mailto:rkbuma@gmail.com)

**Supplementary data D1:**

Raw data files of Salts instrumentations

<https://drive.google.com/open?id=1c6xYyEPJri-ZbZYvvONuuyEWBmwkquFO>

**Supplementary Text T1: Detailed instrumentation results**

1. **Table salt**
2. **GCMS**

**
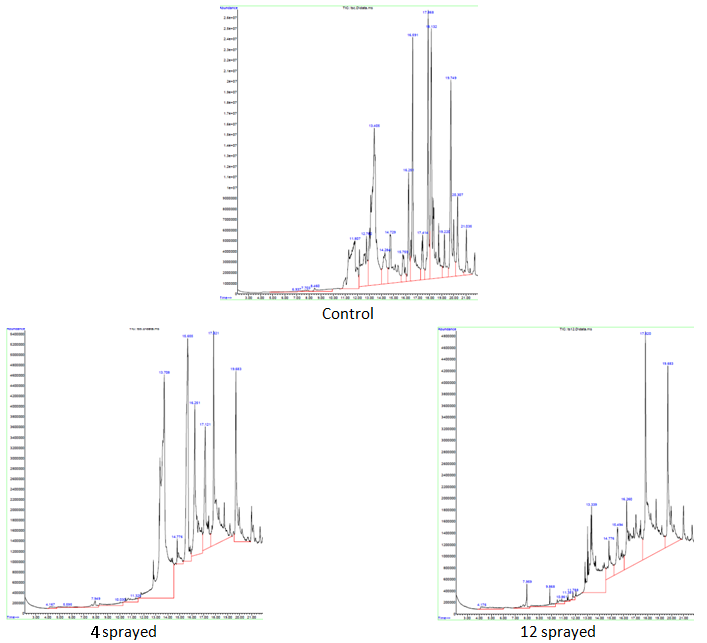
**

**Table salt – GC-MS analysis**

| **Rt (min)** | **Name of compound** | **% area present in each sample** | | | **Remarks** |
| --- | --- | --- | --- | --- | --- |
|  |  | **Control** | **4 sprayed** | **12 sprayed** |  |
| 11.807 | n-Hexadecanoic acid | 9.57 | 0.0 | 0.26 |  |
| 12.762 | Cyclohexane, (1-methylethyl)- | 6.12 | 0.0 | 0.0 |  |
| 13.339 | Heptylcyclohexane | 0.0 | 0.0 | 21.26 |  |
| 13.405 | cis-13-Octadecenoic acid | 19.78 | 0.0 | 0.0 | Most abundant peak in control |
| 13.708 | sec-Butyl (E)-2-methylbut-2-enoate | 0.0 | 39.78 | 0.0 | Most abundant peak in 4 sprayed sample |
| 14.284 | Oleic Acid | 3.32 | 0.0 | 0.0 |  |
| 14.729 | Palmitoyl chloride | 5.67 | 0.0 | 0.0 |  |
| 14.776 | Oleic acid | 0.0 | 0.0 | 4.37 |  |
| 15.655 | Cyclohexane, (1-methylethyl) | 0.0 | 13.71 | 0.0 |  |
| 16.251 | cis-9-Hexadecenal | 3.36 | 0.0 | 19.98 | Most abundant peak in 12 sprayed sample |
| 16.251 | Hexan-3-yl (E)-2-methylbut-2-enoate | 0.0 | 11.16 | 0.0 |  |
| 16.591 | Glycerol 1-palmitate | 9.36 | 0.0 | 0.0 |  |
| 17.121 | 4H-1-Benzopyran, 4,4,5,8-tetramethyl- | 0.0 | 8.00 | 0.0 |  |
| 17.868 | 2-Cyclohexylnonadecane | 7.91 | 0.0 | 0.0 |  |
| 17.820 | Fumaric acid, cis-hex-3-enyl heptadecyl ester | 0.0 | 0.0 | 28.67 | Most abundant peak in 12 sprayed sample |
| 17.821 | Octane, 2-cyclohexyl | 0.0 | 14.46 | 0.0 | Most abundant peak in 4 sprayed sample |
| 18.132 | Oleic acid, 3-hydroxypropyl ester | 14.23 | 0.0 | 0.0 |  |
| 19.220 | Cyclohexane, 1,1'-(1,4-butanediyl)bis- | 2.18 | 0.0 | 0.0 |  |
| 19.683 | 9-Octadecenoic acid (Z)-, 2-hydroxy-1-(hydroxymethyl)ethyl ester | 0.0 | 8.00 | 12.98 |  |
| 19.749 | 9-Octadecenoic acid (Z)-, 2,3-dihydroxypropyl ester | 8.28 | 0.0 | 0.0 |  |
| 20.307 | 6-Octadecenoic acid, (Z) | 3.29 | 0.0 | 0.0 |  |

Table salt is typically 97 – 99 percent sodium chloride (NaCl). Additional compounds in table may include potassium iodide, sodium iodide, sodium iodate, dextrose, sodium fluoride, iron salts (e.g. ferrous fumarate), folic acid (vitamin B9), and anti-caking agents such as calcium aluminosilicate, calcium carbonate, calcium silicate, fatty acid salts, magnesium carbonate, magnesium oxide, silicon dioxide, sodium aluminosilicate, sodium ferrocyanide, and tricalcium phosphate. A combination of NaCl and any and all of the compounds above may give the control sample its normal taste.

Control sample contains cis-13-Octadecenoic acid, n-Hexadecanoic acid, 9-Octadecenoic acid (Z)-, 2,3-dihydroxypropyl ester as major peak which might have derived from the solvent contamination of previous samples used for analysis or GC. After 4 sprayings, there was new peak of sec-Butyl (E)-2-methylbut-2-enoate was come as major peak which might be responsible for enhancement of saltiness characters. While 12 sprayed sample has shown unique peak of Fumaric acid, cis-hex-3-enyl heptadecyl ester which might be responsible for corresponding changes. In addition, there were peaks of Heptylcyclohexane and cis-9-Hexadecenal but these could be result of previous GC sample run on GC column.

1. **FTIR**

**
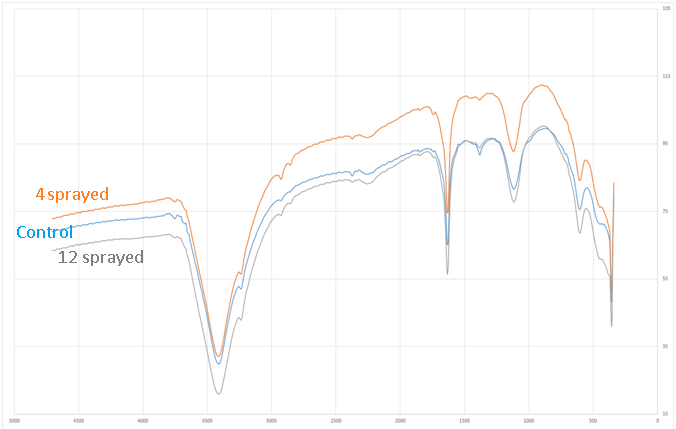
**

Peak 3410, 15cm^-1^ show OH groups water (maybe in crystalohydrates) content in sample. This peak is increased in 12 sprayed sample for 28% than control and 4 sprayed samples. Peak 1635 cm^-1^ show presence of fluorides (HF_2_^-^). In 4 sprayed sample we can see decrease of this peak in 3 % however, in 12 sprayed sample, an increase of this peak by 36% can be noticed, which could cause an unpleasant taste. Peak 1018cm^-1^ show presence of silicates (SO_3_^2-^). In 4 sprayed sample we can see decrease of this peak in 1% and, in 12 sprayed sample, increase of this peak in 26% can be noticed, which could cause an unpleasant taste.

1. **PXRD**

**
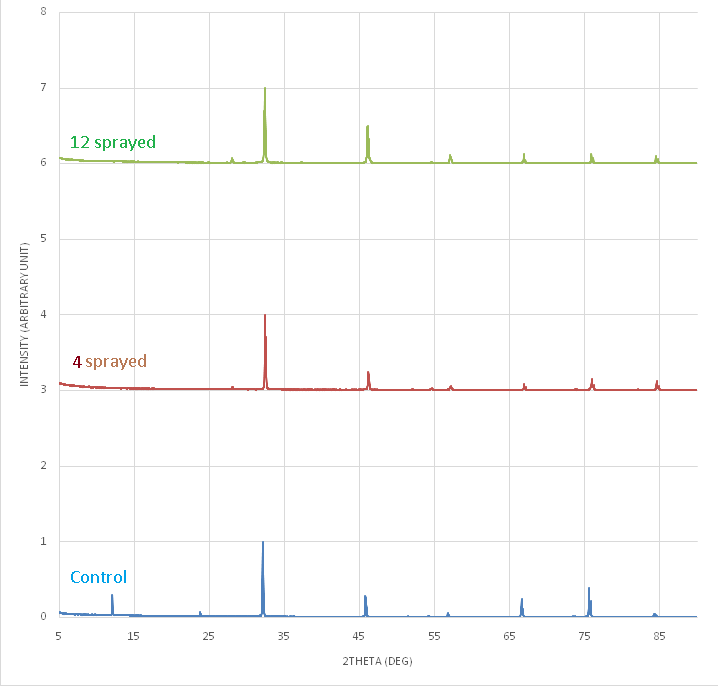
**

**Fig: PXRD Table salt**

**Control**: shows several prominent peaks from 2-theta range12.0 ^o^ to 85.0^o^. The most prominent peak is located at 32.1^o^, followed by the peaks at 75.6^o^, 12.1^o^, 45.8^o^, and 66.6^o^ respectively. Minor peaks are located at 24.1^o^, 57.2^o^, and 84.6^o^. The present peaks indicate the presence of cubic structure, close to the mineral halite (Walker *et al*., 2004; Wang *et al*., 1996; Almeida, 2017; Halite R070292). Peak around 12.0^o^ is rarely observed in NaCl samples. This peak, however, is present in salt crystals with structure NaCl_3_, another stable stoichiometry of NaCl (Zhang *et al*., 2013). The XRD pattern shows good crystalline structure with minimal amorphous phases. Split-peaks are observed in minor peaks 31.1, 45.8^o^, 57.2^o^, 66.6^o^, and 84.6^o^ indicating departure from the standard cubic crystal structure.

**4 sprayed sample**: shows several prominent peaks from 2-theta range 28.0 ^o^ to 85.0^o^. The most prominent peak is located at 32.4^o^, followed by the peaks at 46.2^o^, 75.9^o^, 84.6^o^, and 67.1^o^,respectively. Minor peaks are located at 28.5^o^, 55.1^o^, and 57.4^o^. Split-peaks are observed in minor peaks 57.4^o^, 67.1^o^, and 84.6^o^indicating departure from the standard cubic crystal structure. TS5 shows similar peaks with control, indicating correspondence with halite mineral structure (Walker *et al*., 2004; Wang *et al*., 1996; Almeida, 2017; Halite R070292).

**12 sprayed sample**: shows several prominent peak from 2-theta range 28.0 ^o^ to 85.0^o^. The most prominent peak is located at 32.4^o^, followed by the peaks at 46.1^o^, 76.1^o^, 66.9.6^o^, 57.1^o^, and 84.7^o^respectively. Minor peaks are located at 28.0^o^, 37.7^o^, and 54.^o^. Split-peaks are observed in the most prominent peak at 32.4^o^ and at minor peaks 57.4^o^, 67.1^o^, and 84.6^o^ indicating departure from the standard cubic crystal structure. 12 sprayed sample shows similar peaks with control, indicating correspondence with halite mineral structure (Walker *et al*., 2004; Wang *et al*., 1996; Almeida, 2017; Halite R070292)

Control shows more prominent and minor peaks among the three samples. It also has an NaCl_3_phase present, in contrast with 5 and 12 sprayed samples. Structure of 5 and 12 sprayed samples are closer compared to control, due to the presence of other underlying phases. Varying of peaks intensities are also observed. Peak at around 46.0^o^is highest at 12 sprayed sample, relative to the 100% peak, followed by control, and lastly 4 sprayed. No shifting of peaks is observed among the three samples.

1. **TEM**


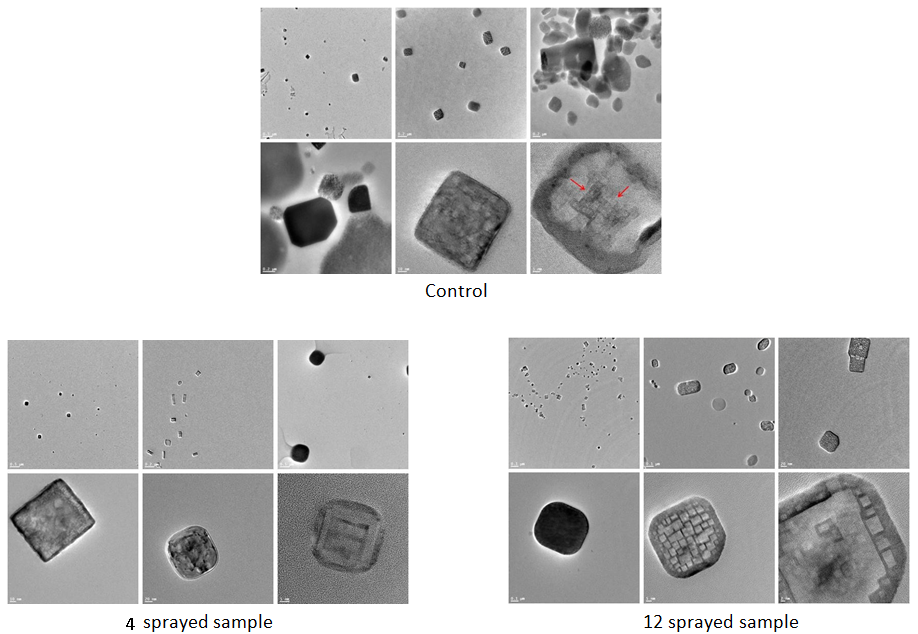


**Control**: Particle size range 100-500 nm and amorphous.

**4 sprayed**: Particle size range 50-200nm and crystal.

**12 sprayed**: Particle size range 10-50nm numerosity appears larger, and crystalline. Interplanar distance has narrower variability range of values indicating an enhanced regular crystalline structure.

Degree of crystallinity and the regularity of the crystal structure are enhanced proportionally with increasing number of MIRGA sprayings. Orientation of crystal lattice planes become increasingly regular and parallel to the incident electron beam, making visible the columns of atoms of the crystal lattice by increasing number of MIRGA sprayings.

1. **Rock salt**
2. **GC-MS**

**
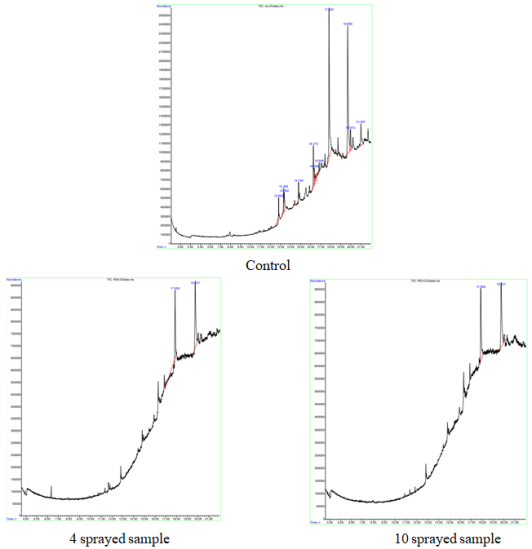
**

**Fig: GC-MS spectra of rock salt samples**

**GC-MS analysis of rock salt samples**

| **R.T. (Min)** | **Name of Compound** | **% Area presence in sample** | | | |
| --- | --- | --- | --- | --- | --- |
|  |  | **Control** | **4 sprayed sample** | **10 sprayed sample** | **Remarks** |
| 12.809 | 13-Hexyloxacyclotridec-10-en-2-one | 1.66 | 0.0 | 0 |  |
| 13.301 | 1-Azabicyclo[3.1.0]hexane | 3.22 | 0.0 | 0.0 |  |
| 13.358 | Pyridine, 2,3,4,5-tetrahydro | 5.07 | 0.0 | 0.0 |  |
| 14.795 | Oleic Acid | 0.61 | 0.0 | 0.0 |  |
| 16.270 | 7-Pentadecyne | 7.25 | 0.0 | 0.0 |  |
| 16.355 | cis-9-Hexadecenal | 2.68 | 0.0 | 0.0 |  |
| 16.847 | Benzene, 1,1'-(2-butene-1,4-diyl)bis | 3.66 | 0.0 | 0.0 |  |
| 17.830 | Octane, 2-cyclohexyl- | 34.33 | 0.0 | 0.0 | *Most abundant present in Control* |
| 17.855 | 13-Octadecenal, (Z)- | 0.0 | 0.0 | 43.84 | *Only present in 10 sprayed sample* |
| 17.855 | Undecylenic acid | 0.0 | 20.97 | 0.0 | *Only present in 4 sprayed sample* |
| 19.727 | Z-8-Pentadecen-1-ol acetate | 0.0 | 0.0 | 56.16 | *Only present in 10 sprayed sample* |
| 19.727 | 6-Octadecenoic acid, (Z)- | 0.0 | 79.03 | 0.0 | *Only present in 4 sprayed sample*  *Antibacterial, anticancer, immune stimulant, antiaging, anti-inflammatory (***Mustapha *et al*., 2016**; **Helioswilton *et al*., 2013**; **European Patent Office, 2000**) |
| 19.693 | 9-Octadecenoic acid (Z)-, 2-hydroxy-1-(hydroxymethyl)ethyl ester | 32.47 | 0.0 | 0.0 | *Second most abundant present in control* |
| 19.976 | 6-Octadecenoic acid | 4.86 | 0.0 | 0.0 |  |
| 21.007 | 9-Octadecenoic acid (Z)-, 2,3-dihydroxypropyl ester | 7.51 | 0.0 | 0.0 |  |

The rock salt sample contains many aldehyde and long chain fatty acids. After four spraying, the saltiness is increased and the long and short chain fatty acids were found. More precisely, the long chain fatty acid (C18, 6-Octadecenoic acid) might have broken down to Medium chain (C11, Undecylenic acid) during spraying. When the rock salt sample was 10 sprayed for reduction of pungent and saltiness, there were found the major peak of Pentadecen-1-ol acetate and 13-Octadecenal. These both could be theby-product of long chain fatty acids degradation and transformation.

**Mustapha N. Abubakar and Runner R. T. Majinda.GC-MS Analysis and Preliminary Antimicrobial Activity of Albiziaadianthifolia (Schumach) and Pterocarpusangolensis (DC). Medicines 2016, 3, 3; doi:10.3390/medicines3010003**

**Helioswilton Sales-Campos, Patricia Reis de Souza, BethaneaCremaPeghini, Joao Santana da Silva, Cristina Ribeiro Cardoso.An Overview of the Modulatory Effects of Oleic Acid in Health and Disease. Mini-Reviews in Medicinal Chemistry, 2013, 13, 000-000**

**European Patent Office. Application number: 99204230.9. Date of publication: 28.06.2000 Bulletin 2000/26**

1. **FTIR**


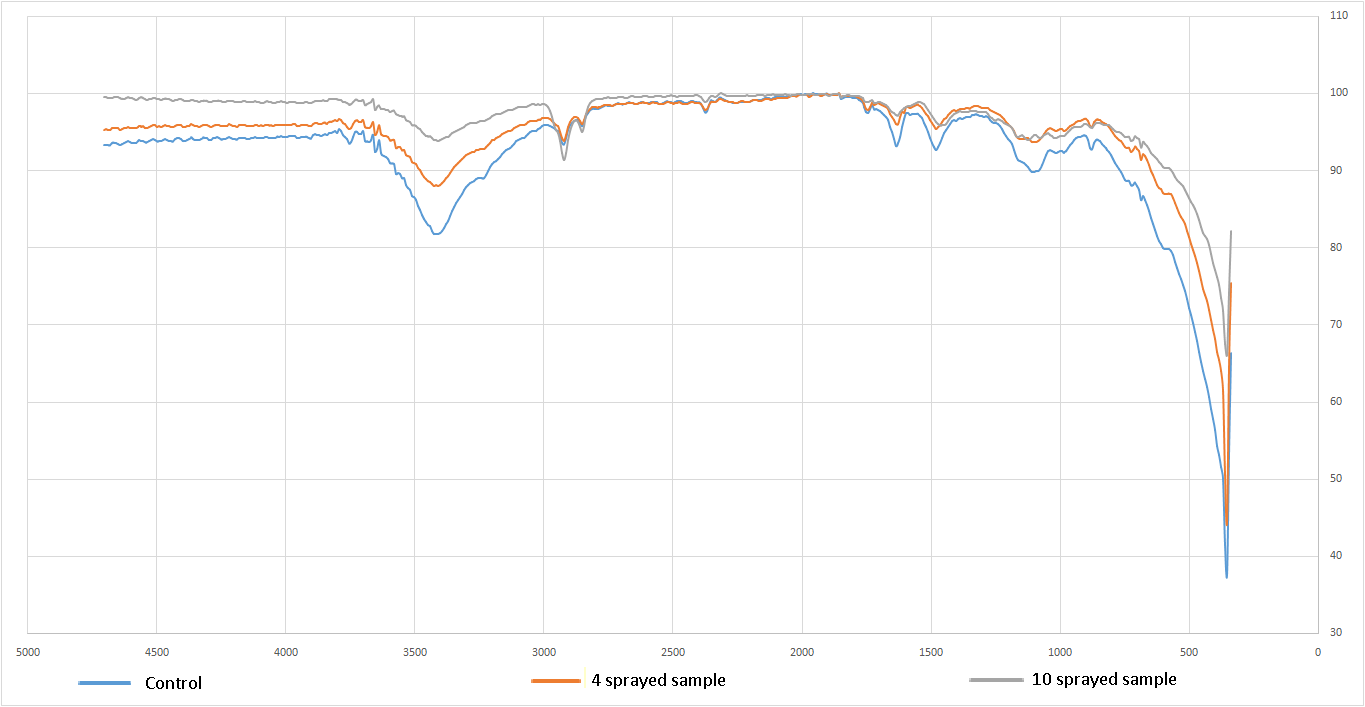


**Fig: FTIR spectra of rock salt samples**

Peak 3410, 15cm^-1^ show water (may be in crystalohydrates) content in sample. In 4 sprayed sample we can see decrease of that peak for 2 times; in 10 sprayed sample we can see decrease of that peak for 2.7 times. Peak 2924 cm^-1^ show presence of bicarbonates. In 4 sprayed sample we can see increase of this peak in 3 % however, in 10 sprayed sample, an increase of this peak by 136% can be noticed, which could cause unpleasant taste. Peak 1627cm^-1^ show presence of fluorides. In 4 sprayed sample we can see decrease of this peak in 46 % and, in 10 sprayed sample decrease of this peak in 48% can be noticed. Peak 1481cm^-1^ show presence carbonates. In 4 sprayed sample we can see decrease of this peak in 35 % and, in 10 sprayed sample, decrease of this peak in 33% can be noticed. Peak 1018cm^-1^ show presence of silicates. In 4 sprayed sample we can see increase of this peak for 5 times and, in 10 sprayed sample, increase of this peak for 7 times can be noticed.

1. **PXRD**


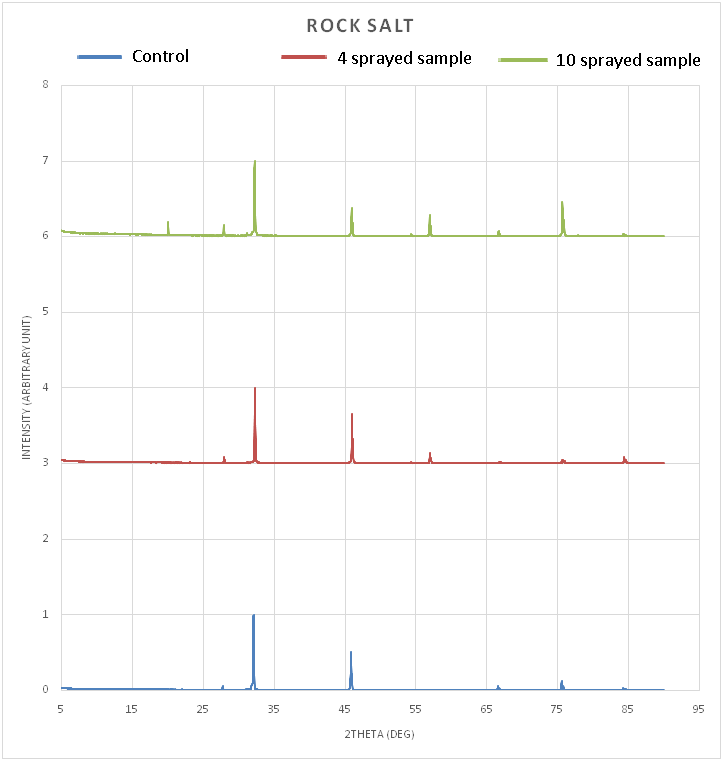


**Fig: PXRD of rock salt**

Control: shows several prominent peaks from 2-theta range 28.0 ^o^ to 85.0^o^. The most prominent peak is located at 32.1^o^, followed by the peaks at 45.9^o^, 75.6^o^, 12.1^o^, and 66.8^o^ respectively. Minor peaks are located at 28.3^o^ and 84.9^o^. The present peaks indicate the presence of cubic structure, close to the mineral halite **(Walker *et al*., 2004,** **Almeida, 2017; RRUFF, Halite R070292)**. The XRD pattern shows good crystalline structure with minimal amorphous phases. Split-peaks are observed in minor peak 75.6^o^indicating departure from the standard cubic crystal structure.

4 sprayed sample: shows several prominent peaks from 2-theta range 28.0 ^o^ to 85.0^o^. The most prominent peak is located at 32.3^o^, followed by the peaks at 46.0^o^and 57.0^o^ respectively. Minor peaks are located at 28.0^o^, 67.2^o^, 76.1^o^, and 84.6^o^. The present peaks indicate the presence of cubic structure, close to the mineral halite **(Walker *et al*., 2004,** **Almeida, 2017; RRUFF, Halite R070292)**. The XRD pattern shows good crystalline structure with minimal amorphous phases. Split-peaks are observed in minor peaks 46.0^o^, 57.0^o^, and 84.6^o^ indicating departure from the standard cubic crystal structure.

10 sprayed sample: shows several prominent peaks from 2-theta range 20.0 ^o^ to 85.0^o^. The most prominent peak is located at 32.3^o^, followed by the peaks at 75.7^o^, 46.0^o^, and 57.0^o^ respectively. Minor peaks are located at 20.1^o^, 28.0^o^, 31.7^o^, 55.2^o^, 67.3^o^, and 85.0^o^. The present peaks indicate the presence of cubic structure, close to the mineral halite **(Walker *et al*., 2004,** **Almeida, 2017; RRUFF, Halite R070292)**. The XRD pattern shows good crystalline structure with minimal amorphous phases. Split-peaks are observed in minor peaks 20.1^o^, 46.0^o^, 57.0^o^, and 75.7^o^indicating departure from the standard cubic crystal structure. Peak around 20.1^o^ is rarely observed in NaCl samples. This peak, however, is present in salt crystals with structure NaCl_3_ that is another stable stoichiometry of NaCl **(Wang and Reeber, 1996)**

Comparison of control, 4 and 10 sprayed sample: 10 sprayed sample has the most number of visible peaks. There are six prominent peaks observed in this sample’s XRD. The peak around 46.0^o^ has highest intensity in 4 sprayed. This is followed by the peaks in control and 10 sprayed sample, respectively. Peak at around 75.0^o^ is highest at 10 sprayed, followed by control and 4 sprayed samples, respectively. Peak at around 28.0^o^ is highest in 10 sprayed, followed by 4 sprayed, and control samples respectively. Peak at around 55.0^o^ is highest in 10 sprayed, followed by in 4 sprayed sample. This peak is absent in the control sample.10 sprayed sample has one more peak at 20.1^o^ which is absent in both control and 4 sprayed samples. Shifting of the most prominent peak to higher 2-theta value is observed in both sprayed samples.

1. **D. Walker, P.K. Verma, L.M.D Cranswick, R.L. Jones, S.M. Clark, and S. Buhre, " Halite-sylvite thermoelasticity,"American Mineralogist, vol. 89, pp. 204-210. 2004**
2. **K.M.F. Almeida, D.M, "Stability field of the Cl-rich scapolite marialite," American Mineralogist, vol. 102, pp. 2484-2493. 2017**
3. **RRUFF, Halite R070292.** [**http://rruff.info/halite/chem=Na,%20Cl/display=default/R070292**](http://rruff.info/halite/chem=Na,%20Cl/display=default/R070292)
4. **K. Wang, R.R. Reeber, "Thermal expansion of alkali halides at high pressure: NaCl as an example," Physics and Chemistry of Minerals, vol 23 pp. 354-360. 1996.**
5. **TEM**


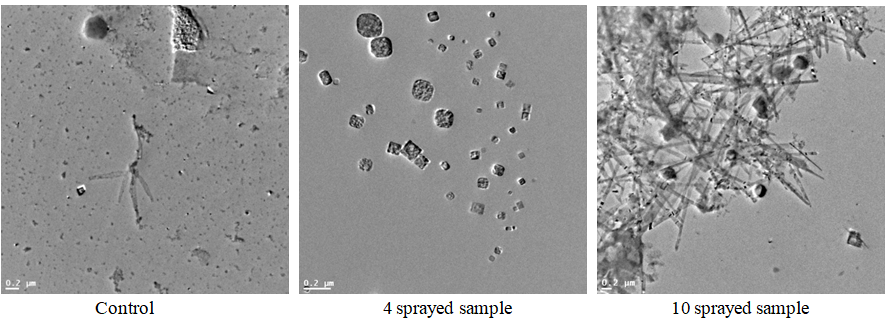


**Fig: TEM bright field images of rock salt**

**
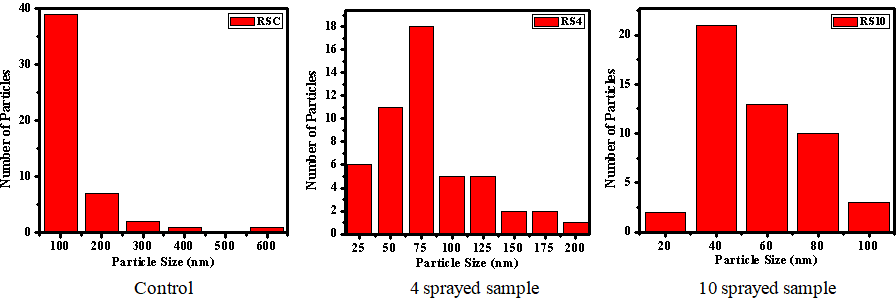
**

**Fig: TEM particle size representation of rock salt samples**

1. **Iodised salt**
2. **GC-MS**

**
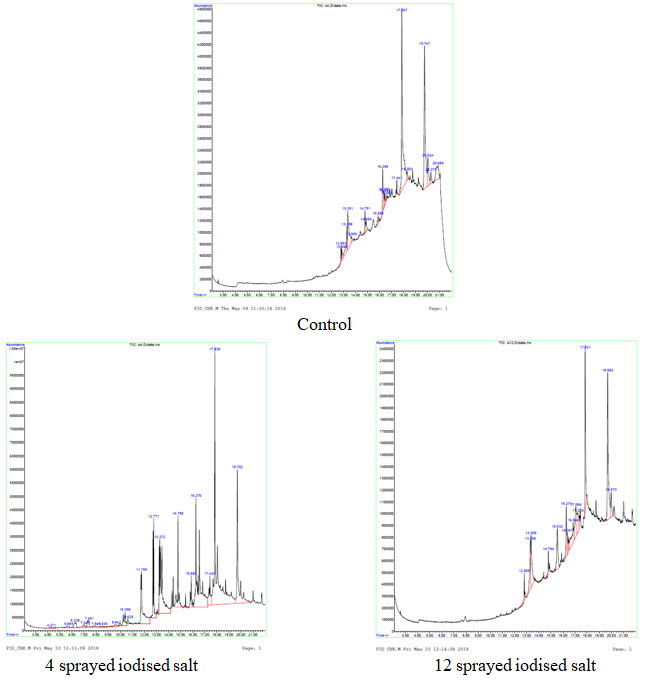
**

**Fig: GC-MS spectra of iodised salt samples**

**GC-MS analysis of Iodised salt**

| **Rt (min)** | **Name of compound** | **% area present in each sample** | | | **Remarks** |
| --- | --- | --- | --- | --- | --- |
|  |  | **Control** | **4 sprayed** | **12 sprayed** |  |
| 11.769 | n-Hexadecanoic acid | 0 | 7.68 | 0 |  |
| 12.771 | 13-Hexyloxacyclotridec-10-en-2-one | 0 | 5.41 | 0 |  |
| 13.301 | 2(3H)-Furanone, 4,5-dihydro-4-(2-methyl-3-methylene-1-buten-4-yl)- | 3.73 | 0 | 0 |  |
| 13.310 | Fumaric acid, trans-hex-3-enyl tridecyl ester | 0 | 0 | 5.42 |  |
| 13.348 | Cyclohexane, octyl- | 9.05 | 0 | 0 |  |
| 13.358 | Cyclopentanone, 3-methyl-2-(2-pentenyl)- | 0 | 0 | 10.91 |  |
| 13.272 | 1,1'-Bicyclohexyl | 0 | 13.86 | 0 |  |
| 14.785 | 1,15-Pentadecanedioic acid | 0 | 4.99 | 0 |  |
| 14.795 | Octadec-9-enoic acid | 2.04 | 0 | 0 |  |
| 15.532 | Dodecane, 2-cyclohexyl- |  | 0 | 8.17 |  |
| 16.270 | Oleic Acid | 4.40 | 0 | 0 |  |
| 16.270 | 13-Octadecenal, (Z)- | 0 | 16.58 |  |  |
| 16.270 | cis-9-Hexadecenal | 0 |  | 6.45 |  |
| 17.423 | Bicyclo[5.3.1]undecan-11-one | 0 | 28.86 | 0 | Most abundant peak in 4 sprayed sample |
| 16.847 | 9-Octadecenoic acid (Z)-, 2,3-dihydroxypropyl ester | 0 | 0 | 6.85 |  |
| 17.064 | 1,2-Benzisothiazole, 3-(hexahydro-1H-azepin-1-yl)-, 1,1-dioxide | 0 | 0 | 7.64 |  |
| 17.225 | 3-(O-Nitrophenyl)-2-oxo-1,3-oxazolidine | 0 | 0 | 5.63 |  |
| 17.830 | 1-Tridecene | 0 | 0 | 16.65 |  |
| 17.858 | Fumaric acid, cis-hex-3-enyl nonadecyl ester | 32.31 | 0 | 0 | Most abundant peak in control |
| 19.702 | Hexan-3-yl (E)-2-methylbut-2-enoate | 0 | 11.64 | 0 |  |
| 19.749 | 9-Octadecenoic acid (Z)-, 2,3-dihydroxypropyl ester | 25.84 | 0 | 24.78 | Most abundant peak in 10 sprayed sample |
| 19.976 | cis-11-Hexadecenal | 0 | 0 | 3.92 |  |
| 20.024 | Octadecanal | 5.01 | 0 | 0 |  |
| 20.865 | Oleic Acid | 5.79 | 0 | 0 |  |

Control sample contains Hexan-3-yl (E)-2-methylbut-2-enoate, 9-Octadecenoic acid (Z)-, 2,3-dihydroxypropyl ester, and Cyclohexane, octyl as major peak. After 4 spraying, there was new peak of Bicyclo[5.3.1]undecan-11-one, 13-Octadecenal and 1,1'-Bicyclohexyl. These might be responsible for enhancement of saltiness characters in 4 sprayed sample. While 12 sprayed sample has shown increase in peak of 9-Octadecenoic acid (Z)-, 2,3-dihydroxypropyl ester might be responsible for corresponding changes. In addition, there were new peaks of 1-Tridecene and Cyclopentanone, 3-methyl-2-(2-pentenyl).

Iodised salt consists of any salt (e.g. table salt) that includes addition of an iodine salt such as potassium iodide.

**Control**: shows major peaks at around 12.8 min, 13.3 min, 14.7 min, 16 min, 17.4 min, 17.8 min, 19.7 min, 20 min, 20.2 min, and 20.9 min. It is suggested the normal taste arises from compounds detected by GCMS.

**4 sprayed sample**: is different that the control sample. The key differences are additional peaks at around 10.4 min, 10.6 min, and 11.8 min. It is suggested these additional peaks may be related to the increased saltiness of the 4 sprayed sample relative to the control sample.

**12 sprayed sample**: There are key additional peaks at 15.5 min and 16.2 min compared to the control sample. These additional peaks may be due to compounds that alter the taste of the 12 sprayed sample making it pungent and undesirable with reduced saltiness.

1. **FTIR**


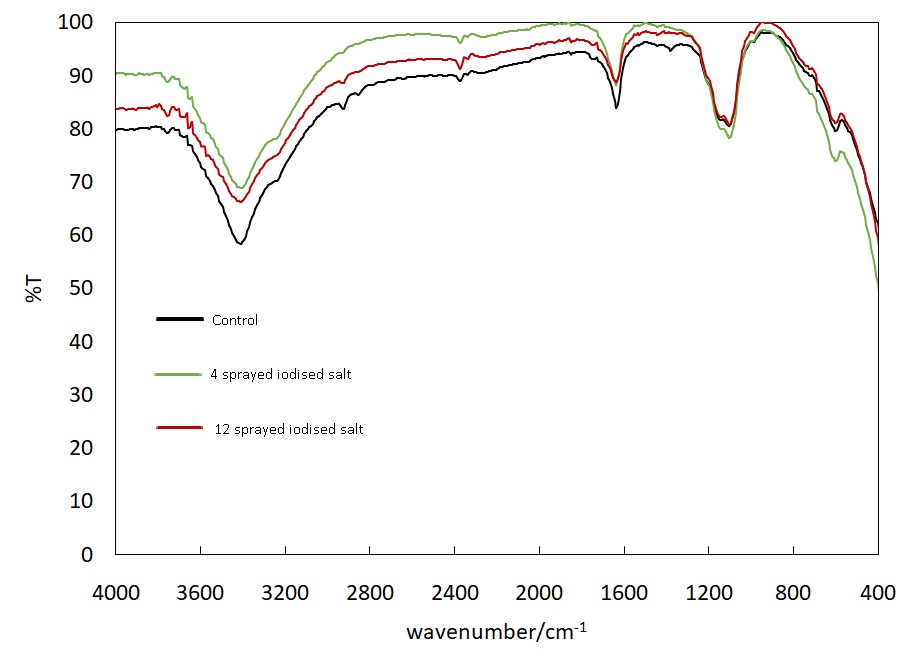


**Fig: FTIR spectra of iodised salt samples**

1. **PXRD**


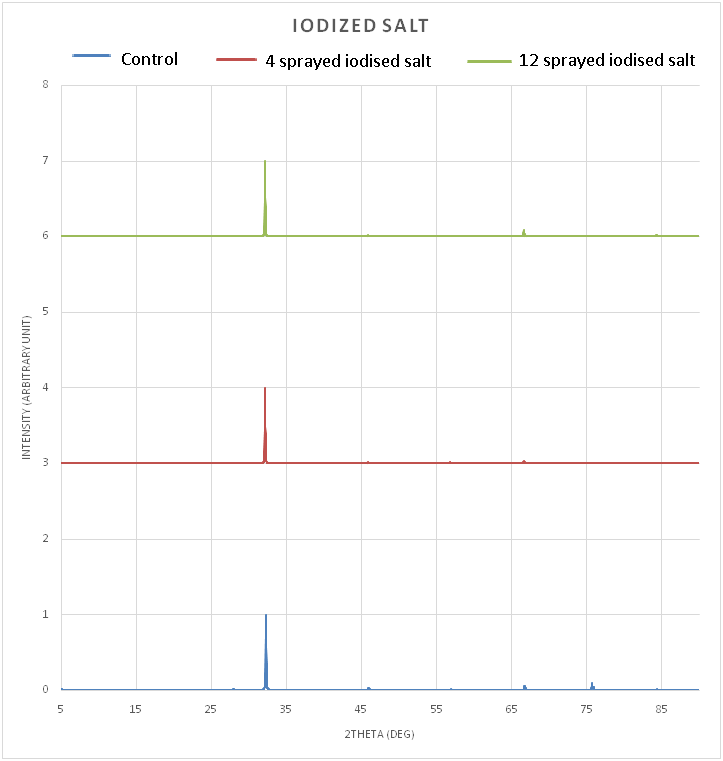


**Fig 5: PXRD of iodised salt**

1. **TEM**


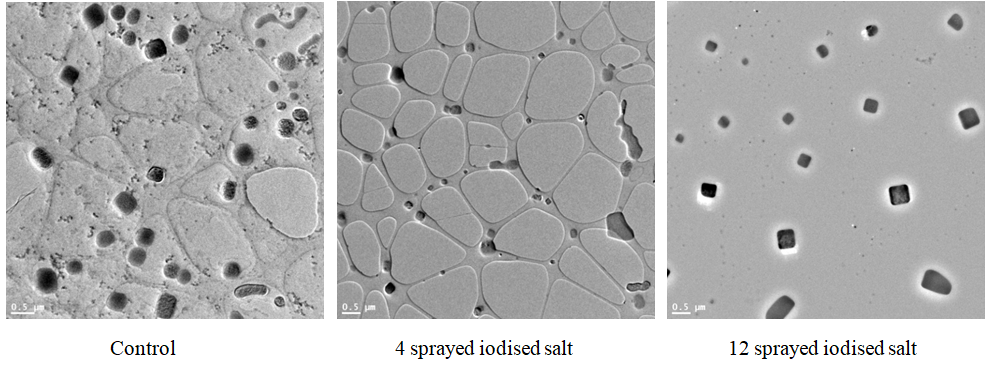


**Fig: TEM bright-field images of iodised salt**

**
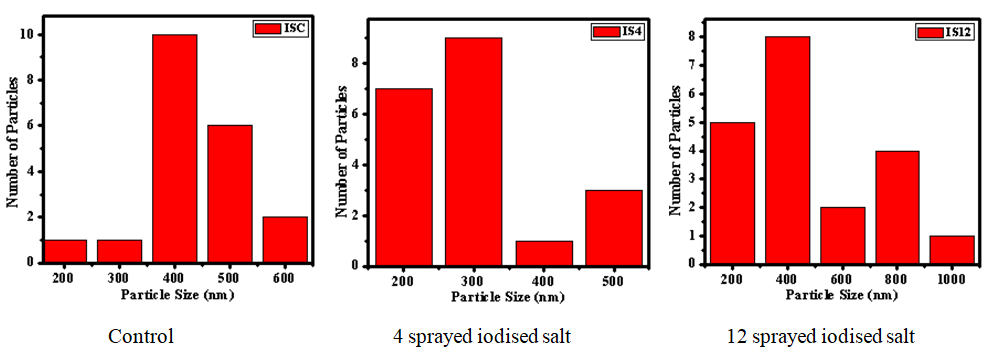
**

**Fig: TEM particle size representation of iodised salt samples**

**Supplementary Text T2: Detailed Discussion**

1. *Detailed discussion [1]*
   1. *Invention background*

The four observable states of matter (solid, liquid, gas, and plasma) are composed of intermolecular and intramolecular bonds. The inherent characteristics of neutrons, protons and electrons are unique, however, differences in their numbers are what constitute different atoms, and how these atoms bind together develops into different molecules with unique characteristics. In the electromagnetic wave (EMW) spectrum, the mid-IR region is vital and interesting for many applications since this region coincides with the internal vibration of most molecules [2]. Almost all thermal radiation on the surface of the Earth lies in the mid-IR region, indeed, 66% of the Sun’s energy we receive is infrared [3] and is absorbed and radiated by all particles on the Earth. At the molecular level, the interaction of mid-IR wavelength energy elicits rotational and vibrational modes (from about 4500–500 cm^-1^, roughly 2.2 to 20 microns) through a change in the dipole movement, leading to chemical bond alterations [4].

During our research we have observed: **(A)** In all objects, even though atoms always remain as atoms, their chemical bond parameters are continuously prone to alteration by cosmic and physical energies (e.g.: EMW, heat, pressure, and humidity) causing the bonds to compress/stretch/bend [5-8], break [9,10], or new bonds to be formed [11]. These alterations ultimately lead to changes in the physicochemical characteristics of the objects. **(B)** The dynamic, constant, and mutual influences of EMW among the Earth and the celestial and living bodies are continuously causing alterations in the inherent physiochemical characters of earthly objects, for instance, enhancement due to an optimum dose of energy or decrease/destruction due to a high dose of energy (detailed below). Thus, based on these concepts, MIRGA was developed to alter the bond parameters, thereby potentiating the natural characteristics of products.

- 1. *MIRGA definition*

We define MIRGA as *‘a harmless, economical atomizer containing an imbalanced ratio of ions suspended in water, which influence the natural potency of target substances by generating mid-IR while spraying’*.

- 1. *Technique of mid-IR generation from MIRGA*

We designed MIRGA as to accommodate an imbalanced ratio of ions suspended in water in their fundamental state, which can move as free particles. The solution exhibits very little detectable background frequency, below even that of cosmic events. By comparison humans emit more radioactivity (around 10 microns) [12,13]. We designed MIRGA to generate energy based on various processes such as:**(A)** spraying leads to ionization (electrons getting separated from atoms) and many pathways for electron re-absorption; due to these two oscillatory processes, energy is generated;**(B)** while spraying, a water-based ionic solution gets excited/charged, which in turn leads to oscillation among the imbalanced ions [14] in their excited state, resulting in the emission of photons [15,16];**(C)**although a low electromagnetic field exists between the charged particles of the MIRGA’s ionic solution, during spraying the induced oscillation between these charged particles produces energy [17-21]; and**(D)** in the natural rainfall process, more energy is required to break the water bonds for creating smaller water droplets [22]. Therefore, these droplets should have more stored energy, which then travels down at velocity from a specific distance, thus gaining kinetic energy. When the rain hits the Earth’s surface, it forms a very thin film of mid-IR (nearly 6 micron), hence there is a net heat gain [22,23]. We simulated this rainfall’s energy-gaining process in MIRGA(i.e., when imbalanced ions in liquid media are atomized, the ejected smaller droplets should have higher internal energy as well as acquired kinetic energy, and the energy emitted by breaking the surface tension). From trial and error, we calibrated the ejection pressure to obtain a desired fine mist, and minimized the evaporation rate by altering the pH and density of the solution. Moreover, the accelerated ions in the sprayed ionic clouds collide among themselves and generate energy [24], thus, we incorporated these phenomena in our atomizer and designed it in such a way as to emit energy in the 2–6 µm mid-IR depending on the given plunger pressure.

Yousif et al. [25] described this process as a photodissociation of molecules caused by the absorption of photons from sunlight, including those of infrared radiation, visible light, and ultraviolet light, leading to changes in the molecular structure.

- 1. *Safety of MIRGA-sprayed products*

In our nearly two-decades of research, we have observed that MIRGA-induced bond-altered target substances do not show any adverse reaction upon consumption/use. In nature, **(A)** Stereochemical configuration has great influence on taste [26] (e.g., varieties of mango, grapes, rice, etc.), **(B)** Cooking and digestive enzymes break chemical bonds, thereby softening foods. This indicates that alterations in chemical bonds occur naturally and do not represent a risk to human health. As an example, boiled rice, puffed rice, flat rice, and rice flour have a unique aroma, taste, texture, and shelf-life but conserving the same molecular formula (C_6_H_10_O_5_).**(C)** In the food industry, sensory attributes and shelf-life are enhanced by altering the food’s chemical bonds using various irradiation processes like radappertization, radicidation, and radurization [27].**(D)** Upon heating, water changes from ice to liquid to steam, which are manifestations of changes in the hydrogen bonds [28] but the chemical composition (H_2_O) remains the same [29].

- 1. *MIRGA’s primeval and future scope*

The water-based MIRGA could be the first novel potentiating technology. This type of atomizer technology also seems to be present with the extra-terrestrials for their therapeutic use during visitations [30].

In various products, we have achieved a range from 30% to 173% potentiation. Even the smaller improvement resulted in 30% monetary and resource savings as well as health benefits. However, there is a knowledge gap between potentiation from 30% to at least 100% for all products, which can be filled-up by refining MIRGA’s ionic solution, concentration, atomizer pressure, and other parameters and even formulating a better solution.

Various mid-IR emitters are now available (e.g., silicon photonic devices [31], cascade lasers quantum and interband [32], non-cascade-based lasers, chalcogenide fiber-based photonic devices [33], and suspended-core tellurium-based chalcogenide fiber photonic devices [34]). These emitters are not as cost-effective as MIRGA and are useful only in astronomy, military, medicine, industry, and research applications. These emitters are too complex for domestic application by the average user.

Because of MIRGA’s wide range of applications, we believe that this technique will resonate in many scientific fields including biophotonics, therapeutics, health, ecology, and others. We are currently conducting research on MIRGA and its applications, namely MIRGA salt, MIRGA vapor and MIRGA plasma.

**References**

1. Umakanthan, Mathi M, 2022. Decaffeination and improvement of taste, flavor and health safety of coffee and tea using mid-infrared wavelength rays. Heliyon, e11338, Vol 8(11). doi: 10.1016/j.heliyon.2022.e11338
2. CORDIS, European commission. New advances in mid-infrared laser technology, Compact, high-energy, and wavelength-diverse coherent mid-infrared source. Available at: <https://cordis.europa.eu/project/rcn/99977/brief/en> (last accessed on 27.01.2019)
3. A. Salam, A. Ammar, A.H. Asaad, L. Yi-Chen, C. Francesco, 2019. Molecules A Comprehensive Review on Infrared Heating Applications in Food Processing. Molecules. 24, 2-21. doi: 10.3390/molecules24224125.
4. J. E. Girard, Principles of Environmental Chemistry, third ed., Jones & Bartlett Learning, USA, 2014, pp. 99.
5. James E. Girard, 2014. Principles of Environmental Chemistry, 3rd edition, Jones & Bartlett Learning, USA, p99
6. Avelion Alvarez and Miguel Prieto, 2012. Fourier Transform Infrared spectroscopy in Food Microbiology, Springer Science & Business Media, p3.
7. Brian C. Smith. Infrared Spectral Interpretation: A Systematic Approach, CRC Press, LLC, 7, (1999)
8. Dwivedi Ravi Shankar, 2017. Remote Sensing of Soils. Germany: Springer-Verlag GmbH, p268
9. Jag Mohan. Organic Spectroscopy: Principles and Applications, 2nd edition, Alpha science international Ltd., Harrow, UK, 19, (2004). Available at: <https://books.google.co.in/books?id=fA08Uy5DR0QC&printsec=frontcover&dq=Jag+Mohan.+Organic+Spectroscopy:+Principles+and+Applications&hl=en&sa=X&ved=0ahUKEwjHpcHUi9fgAhXXFIgKHXvRCpIQ6AEIKjAA#v=onepage&q=Jag%20Mohan.%20Organic%20Spectroscopy%3A%20Principles%20and%20Applications&f=false>
10. Carolyn McMakin, 2011. Frequency specific Microcurrent in pain management E-book, Elsevier, China, p 30.
11. David Moss, 2011. Biomedical Applications of Synchrotron Infrared Microspectroscopy: A Practical Approach, Royal Society of Chemistry, UK, p 58.
12. Peter H. Raven, Linda R. Berg, David M. Hassenzahl, 2012. Environment, John Wiley & Sons, Inc., USA, p45. Available at: <https://books.google.co.in/books?id=QVpO2R51JBIC&pg=RA1-PA45&dq=electromagnetic+waves+make+form+new+bonds&hl=en&sa=X&ved=0ahUKEwiTnO2amMbjAhUJ3o8KHSfkAJEQ6AEIMjAB#v=onepage&q=electromagnetic%20waves%20make%20form%20new%20bonds&f=false>
13. Frances Ashcroft, 2000. Life at the Extremes: The Science of Survival, University of California Press, California, p122
14. Robert H. Sanders, 2014. Revealing the Heart of the Galaxy, Cambridge University Press, USA, p70
15. Frank Verheest. Waves in Dusty Space Plasmas, Kluwer Academic Publishers, Netherlands, 89, (2000)
16. Sun Keping, Gefei Yu. Recent developments in Applied Electrostatics (ICAES2004): Proceedings of the Fifth International Conference on Applied Electrostatics, Elsevier Ltd., UK, p87.
17. Pierre L. Fauchais, Joachim V.R. Heberlein, Maher I. Boulos. Thermal Spray Fundamentals From Powder to Part. Springer Science & Business Media, New York, 84 (2014)
18. Manfred Wendish, Jean-Louis Brenguier. Airborne Measurements for environmental Research: Methods and Instruments, Wiley-VCH. Available at: <https://books.google.co.uk/books?id=tHdwhn-c5mgC&pg=PT419&dq=A+regularly+oscillating+charge+produces+a+harmonic+electromagnetic+waves+Manfred&hl=en&sa=X&ved=0ahUKEwjBqdv75tvgAhWpSxUIHbQ_D0gQ6AEIKjAA#v=onepage&q=A%20regularly%20oscillating%20charge%20produces%20a%20harmonic%20electromagnetic%20waves%20Manfred&f=false> (last accessed on 27.02.2019)
19. Kongbam Chandramani Singh, 2009. Basic Physics, PHL Learning Private Limited, New Delhi, p413
20. Mathura Prasad. Soul, God and Buddha in Language of Science, Notion Press, Chennai(2017)
21. Stephen Pople, 1999. Complete Physics, Oxford University Press, Oxford, p166
22. Roger Barry, Richard Chorley, 1998. Atmosphere, Weather and Climate, 7th edition, Routledge, London, p51
23. Eniday: <https://www.eniday.com/en/sparks_en/harnessing-the-energy-of-rain/> (last accessed on 06.02.2019)
24. Krishnakumar, T (2019). Application Of Microwave Heating In Food Industry. 10.13140/RG.2.2.27035.72488.
25. Yousif, E., & Haddad, R. (2013). Photodegradation and photostabilization of polymers, especially polystyrene: review. SpringerPlus, 2, 398. <https://doi.org/10.1186/2193-1801-2-398>
26. Kenneth L., Williamson, Katherine M. Masters, 2011. Macroscale and Microscale Organic Experiments, 6th edition, Brooks/ Cole C engage learning, CA, p720
27. Sivasankar B. Food Processing and preservation, PHI Learning Private Limited, Delhi, 246, (2014)
28. Trevor Day, 1999. Ecosystems: Oceans. Routledge Taylor & Francis Group, London and New York, p44
29. Kenneth W. Raymond, 2010. General Organic and Biological Chemistry, 3rd edition, John Wiley & Sons, Inc., USA, p176
30. Blue planet project: Alien Technical research–25, Westchester Camp, Office of the Central Research #3.CODE: ARAMISIII–ADR3-24SM, p80-81
31. CMOS Emerging Technologies. CMOSET 2012: Abstracts, p49. Available at: <https://books.google.co.in/books?id=3XVYC-yBgksC&pg=PA49&dq=mid+infra#v=onepage&q&f=false>
32. Jung, D., Bank, S., Lee, M. L., & Wasserman, D. (2017). Next-generation mid-infrared sources. Journal of Optics, 19(12), 123001. doi:10.1088/2040-8986/aa939b.
33. Sincore, A. & Cook, Justin & Tan, Felix & El Halawany, Ahmed & Riggins, A. & McDaniel, S. & Cook, G. & Martyshkin, Dmitry & Fedorov, V.V. & Mirov, Sergey & Shah, L. & Abouraddy, A. & Richardson, M. & Schepler, Kenneth. (2018). High power single-mode delivery of mid-infrared sources through chalcogenide fiber. Optics Express, 26(6), 7313. doi:10.1364/oe.26.007313.
34. Wu, Bo & Zhao, Zheming & Wang, Xunsi & Tian, Youmei & Mi, Nan & Chen, Peng & Xue, Zugang & Liu, Zijun & Zhang, Peiqing & Shen, Xiang & Nie, Qiuhua & Dai, Shaocong & Wang, R.P. (2018). Mid-infrared supercontinuum generation in a suspended-core tellurium-based chalcogenide fiber. Optical Materials Express, 8(5), 1341. doi:10.1364/ome.8.001341.
